# Impact of SARS-CoV-2 Infection During Pregnancy on Infant Neurobehavioral Development: A Case-Control Study

**DOI:** 10.3389/fped.2021.762684

**Published:** 2021-12-02

**Authors:** Yao Cheng, Haoyue Teng, Yue Xiao, Mengxin Yao, Jieyun Yin, Guoqiang Sun

**Affiliations:** ^1^Department of Obstetric, Maternal and Child Health Hospital of Hubei Province, Wuhan, China; ^2^Department of Epidemiology and Health Statistics, Medical College of Soochow University, Suzhou, China

**Keywords:** COVID-19, pregnancy, infant neurobehavioral development, ASQ-3, SARS-CoV-2

## Abstract

**Background:** Previous studies on the pneumonia outbreak caused by the severe acute respiratory syndrome coronavirus 2 (SARS-CoV-2) have focused on the general population and pregnant women, while little is known about the effects of SARS-CoV-2 on retardation during and after pregnancy. The purpose of this study was to evaluate the potential influence of SARS-CoV-2 on infant neurobehavioral development.

**Methods:** A case-control study was conducted in Maternal and Child Health Hospital of Hubei Province. Nine pregnant women with SARS-CoV-2 infection and 9 controls matched by maternal age, parity, and status of chronic disease were included. Infantile neurobehavioral development was assessed through the Ages and Stages Questionnaires Edition 3 (ASQ-3).

**Results:** The majority of pregnant women with SARS-CoV-2 experienced cesarean section (7 of 9), which was higher than the control group (5 of 9). The throat swabs of all newborn were negative. We found that compared with the control group, neonates of mothers with SARS-CoV-2 infection during pregnancy had lower scores in communication, gross movement, fine movement, problem solving, and personal-social domains; but only fine movement domain yielded statistical significance (*P* = 0.031).

**Conclusion:** Infection with SARS-CoV-2 during pregnancy may have a certain impact on infant neurobehavioral development. Further studies with larger sample size are warranted for validation.

## Introduction

The current pneumonia outbreak of coronavirus disease 2019 (COVID-19) caused by the severe acute respiratory syndrome coronavirus 2 (SARS-CoV-2), is a highly infectious disease. Importantly, on March 11, 2020, World Health Organization (WHO) announced that the ongoing outbreak of COVID-19 virus had become a global pandemic (1, 2). However, nobody can reliably predict how long the COVID-19 pandemic will last.

Pregnant women are a special group and are at higher risk for acquiring infection and dying compared with nonpregnant women (3, 4). Because of the immune and physiological changes that accompany pregnancy, pregnant women are more likely to encounter a series of negative emotions during the pandemic (5). In addition, the measures to deal with COVID-19 (such as isolation, physical distance, home isolation, and remote consultation with health professionals, etc.) may result in the inability to obtain the expected level of support and care before delivery, which may increase the psychological pressure of pregnant women (6, 7). Furthermore, studies have found that SARS-CoV-2 has direct neuroinvasion ability (8), which may attack the human central nervous system and have a potential impact on the survivors' cognitive and neuropsychological functions (9, 10). Mother and baby are closely linked during pregnancy, it is widely believed that the maternal psychological stress, as a teratogen, will continuously transmit painful signals to the unborn child, which may cause adverse effects on the fetus (11). Moreover, early mother-infant separation due to mandatory or voluntary isolation after childbirth may also have a negative impact on infant feeding and thereby early development (12–14). At present, there is little research evidence showing the long-term effects of SARS-CoV-2 infection on pregnant women and their babies. Research is needed to evaluate the burden of SARS-CoV-2 infection during pregnancy in children's personality formation and neurobehavioral development.

Above all, this study aims to evaluate the effect of maternal SARS-CoV-2 infection on the neurobehavioral development of their babies, so as to improve the preventive behavior and health care strategies for pregnant women, new mothers and babies during the COVID-19 pandemic.

## Methods

### Study Population

**Figure 1:**
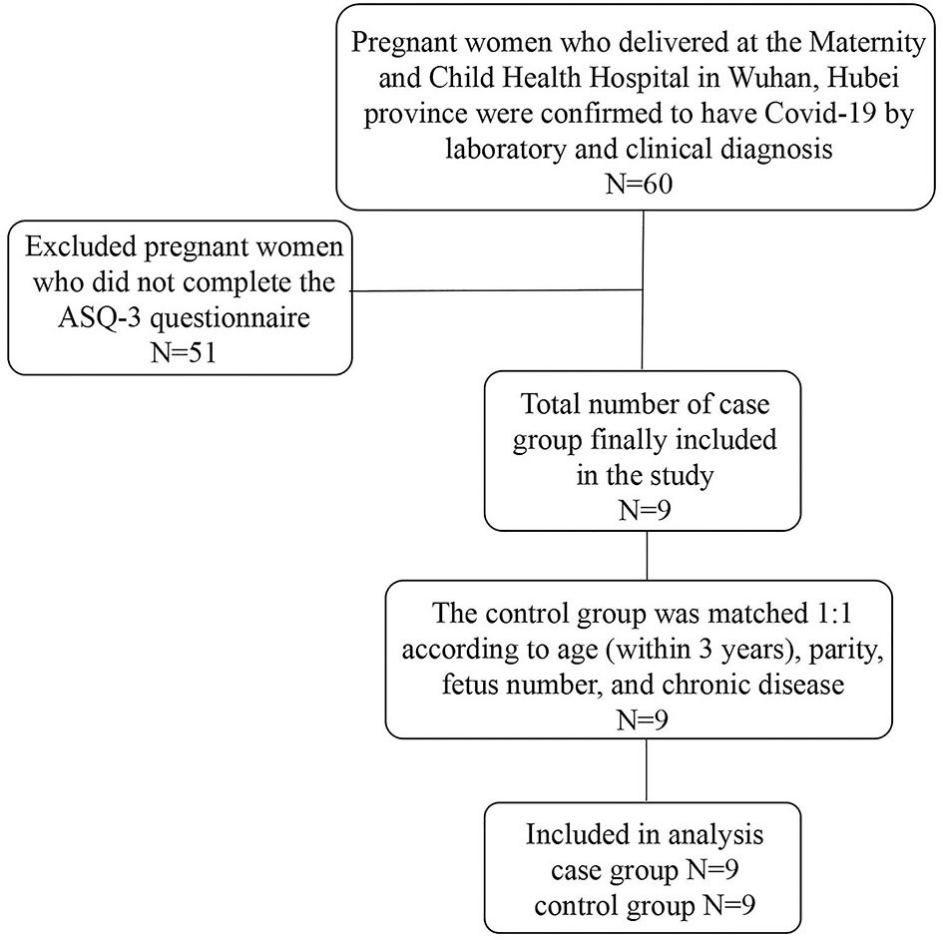
Flowchart of the case-control samples in this study.

A total of 60 patients delivered at the Maternal and Child Health Hospital of Hubei province from January 24 to March 14, 2020, and were confirmed to have COVID-19 by laboratory and clinical diagnosis. The maternal and fetal characteristics of the 60 patients in this study were shown in Supplementary Table 1. Later, 51 patients who refused to finish the Ages and Stages Questionnaires Edition 3 (ASQ-3) questionnaire were excluded. Finally, a total of 9 patients were included in the present analysis. Subsequently, we matched 9 pregnant women without SARS-CoV-2 infection and delivered at the same period at a ratio of 1:1 according to maternal age (within 3 years), parity, and status of chronic disease (including diabetes, hypertension, hypothyroidism, and hepatic disease). All research subjects were recruited in the third trimester. Sociodemographic information and blood samples were collected upon admission. The flowchart of the exclusion and inclusion process of our study population is presented in Figure 1. This study was reviewed and approved by the Ethics Committee of Maternal and Child Health Hospital of Hubei Province (No. 2021IECLW013).

### Neurobehavioral Assessment in Neonates

ASQ-3 is a parent-rated screening tool for the delay of child early development at different ages and stages. ASQ-3 contains 21 questionnaire series, covering all children from 1 month to 5 and a half years old. The children in our study were between 8 and 10 months old when they were surveyed. The ASQ-3 subscale contains 30 items divided into five developmental domains (six items per domain): communication, gross movement, fine movement, problem solving, and personal-social. Each item is rated to a score of 0, 5, and 10, with a total score for each domain of ASQ-3 ranging from 0 to 60 scores (15). There is also a comprehensive question section, which is about the concerns that parents may have about their children. A higher score indicates a higher developmental level for children at each month, and “developmental delay” is defined as a child whose ASQ-3 score is lower than the critical point of their age in any of the five ASQ-3 areas (16). We contacted the parents of the children by phone, and explained the contents of the ASQ-3 questionnaire and the matters needing attention to fill in. An online questionnaire was completed by the child's guardian to obtain an ASQ-3 score.

### Data Collection

The clinical symptoms and signs, laboratory findings and radiologic assessments were extracted from electronic medical records. Two experienced clinicians reviewed and abstracted the data. Maternal respiratory swabs were collected and tested for SARS-CoV-2 by reverse transcriptase–polymerase chain reaction (RT-PCR) assay (17). Positive confirmatory cases of SARS-COV-2 infection were defined as a case where any laboratory test results were positive.

### Statistical Analysis

Continuous and categorical variables were presented as mean ± standard deviation (SD), median [interquartile range (IQR)] and frequency (percentage) when suitable. Continuous variables were compared between two groups by using 2-tail Student's *t*-test or Mann-Whitney's *U*-test when appropriate, and categorical variables were compared by using chi-square test. All statistical analyses were performed using SAS version 9.4. A two-tailed *P* < 0.05 was considered as statistically significant.

## Results

### Maternal Characteristics and Outcomes

The maternal characteristics of cases are shown in Table 1. COVID-19 pregnancies were 31.8 ± 4.6 years old (vs. 31.0 ± 4.9 years old in the control group), and their gestational age at delivery was 38.0 ± 1.1 weeks (vs. 38.9 ± 1.1 weeks in the control group). Besides, the median number of gravidity was 3 in pregnancies with COVID-19, significantly higher than the control group (*P* = 0.003).

**Table 1:** Characteristics of mother-infant pairs with and without SARS-CoV-2 infection.

**Variables**	**Mothers with infection (*n* = 9)**	**Mothers without infection (*n* = 9)**	*P*-value^*^
**Characteristics of pregnant women**			
Age (years)	31.8 ± 4.6	31.0 ± 4.9	0.733
Height (cm)	162.0 ± 4.8	159.8 ± 5.8	0.408
Weight (kg)	57.0 ± 4.4	53.8 ± 4.3	0.153
BMI (kg/m^2^)	21.8 ± 2.0	21.1 ± 1.8	0.499
Gravidity	3 (3, 4)	1 (1, 1)	**0.003**
Parity	1 (1, 2)	1 (1, 3)	0.169
Gestational age (week)	38.0 ± 1.1	38.9 ± 1.1	0.454
Delivery			0.317
Vaginal delivery	2 (of 9)	4 (of 9)	
Caesarean section	7 (of 9)	5 (of 9)	
Gestational hypertension	2 (of 9)	0 (of 9)	0.134
Gestational diabetes	1 (of 9)	2 (of 9)	0.527
**Infantile characteristics**			
Male	3 (of 9)	4 (of 9)	0.629
Height (cm)	49.2 ± 2.1	50.0 ± 1.3	0.362
Weight (g)	3108.8 ± 644.7	3394.4 ± 509.9	0.313
Low birth weight (<2,500 g)	2 (of 9)	0 (of 9)	0.134
Pre-term delivery (<37 weeks)	2 (of 9)	0 (of 9)	0.134
Birth defects	0 (of 9)	0 (of 9)	…
Severe neonatal asphyxia	0 (of 9)	0 (of 9)	…
Neonatal death	0 (of 9)	0 (of 9)	…
Fetal death or stillbirth	0 (of 9)	0 (of 9)	…
Apgar 1 min	9.8 ± 0.4	10.0 ± 0.0	** <0.001**

* *Bold value: P value <0.05 was considered as statistically significant*.

All COVID-19 patients were detected with SARS-CoV-2 infection in the third trimester and presented with mild infection. None of the patients had suffered diabetes, chronic hypertension, or cardiovascular disease before pregnancy. Seven of the nine patients had cesarean section, more than the control group (five of nine). As shown in Table 2, all COVID-19 patients were in stable condition throughout the pregnancy, with body temperature fluctuating within a range of 37.0–38.6°C.

**Table 2:** Detailed characteristics of 9 pregnant women with SARS-CoV-2 infection.

**Case No**.	**1**	**2**	**3**	**4**	**5**	**6**	**7**	**8**	**9**	***n* (%)**
**Clinical characteristics**										
When pneumonia was first detected	31 weeks, 5 days	38 weeks, 6 days	37 weeks, 5 days	40 weeks, 2 days	34 weeks, 4 days	36 weeks, 3 days	42 weeks, 6 days	39 weeks, 1 days	41 weeks, 2 days	...
Symptoms during pneumonia	Prenatal fever cough	Postpartum fever	Postpartum fever	Postpartum fever	Prenatal fever	Prenatal fever	Asymptomatic	Asymptomatic	Postpartum fever	...
Maximum body temperature during pneumonia (°C)	37.7	38.6	38.0	38.4	37.3	37.2	37.0	37.0	37.3	...
Whether to cure at the time of delivery	Yes	No	No	No	No	No	No	No	No	1 (11.1%)
Clinical classification of pneumonia	Mild	Mild	Mild	Mild	Mild	Mild	Asymptomatic	Asymptomatic	Mild	...
**CT evidence of pneumonia**										
Ground-glass shadow	Yes	Yes	Yes	No	No	No	Yes	No	Yes	5 (55.6%)
Unilateral	No	No	Yes	No	No	No	Yes	No	No	2 (22.2%)
Bilateral	Yes	Yes	No	No	No	No	No	No	No	2 (22.2%)
Pleural effusion	No	No	Yes	Yes	No	Yes	No	No	No	3 (33.3%)
Pleural hypertrophy	No	No	No	No	Yes	No	No	No	Yes	1 (11.1%)
Fibrotic lesions	No	No	No	No	No	No	No	Yes	No	1 (11.1%)
**Delivery**										
Delivery mode	Natural vaginal delivery	Cesarean section	Cesarean section	Cesarean section	Cesarean section	Cesarean section	Cesarean section	Cesarean section	Natural vaginal delivery	…
Treatment after delivery										
Antibacterial drugs	Yes	Yes	Yes	Yes	Yes	Yes	Yes	Yes	Yes	9 (100.0%)
Antiviral	No	Yes	No	No	No	No	No	No	Yes	2 (22.2%)
Chinese patent medicine	No	Yes	No	No	No	No	No	No	Yes	2 (22.2%)
Hormones	Yes	Yes	No	No	No	No	No	No	No	2 (22.2%)
Non-invasive oxygen support	Yes	Yes	No	Yes	Yes	Yes	Yes	Yes	Yes	8 (88.9%)
Invasive oxygen support	No	No	No	No	No	No	No	No	No	0 (00.0%)

### Infant's Characteristics and Neurobehavioral Development

As shown in Table 1, all live birth newborn throat swabs in our study were negative. The proportion of premature birth and low birth weight among the newborns of pregnant women with COVID-19 were slightly higher than the control group. Meanwhile, the 1-min Apgar score of the affected group was 9.8 ± 0.4, which was lower than that of the control group (10.0 ± 0.0, *P* < 0.001).

We found that compared with normal pregnant women, neonates of pregnant women with SARS-CoV-2 had lower scores in communication, gross movement, fine movement, problem solving, and personal-social domains. However, only the difference of the fine movement domain reached statistically significant (*P* = 0.031, Table 3).

**Table 3:** The infantile ages and stages questionnaires edition 3 (ASQ-3) scores of mothers with and without SARS-CoV-2 infection.

**Neurobehavioral development [median (IQR)]**	**Mothers with infection (*n* = 9)**	**Mothers without infection (*n* = 9)**	*P*-value^*^
Communication	43 (40,55)	56 (55,60)	0.086
Personal-social	48 (45,55)	51 (50,55)	0.430
Gross movement	42 (30,55)	47 (40,60)	0.521
Fine movement	49 (45,55)	56 (50,60)	**0.031**
Problem solving	51 (45,60)	54 (50,60)	0.401

* *Bold value: P value <0.05 was considered as statistically significant*.

## Discussion

This is a case-control study comparing the neurobehavioral development of neonates in pregnant women with and without COVID-19. It is worth noting that, compared with the control group, the newborns of COVID-19 patients have lower scores in the five developmental areas of ASQ-3, and the difference in fine movement domain is statistically significant.

Pregnant women with COVID-19 were more likely to have babies delivered preterm or with low birth weight, which were strongly associated with delayed cognitive and motor development in infants (18). Motor skills in early infancy can predict the development of infants' subsequent communication ability, and also reflect the strength of learning ability to a certain extent (19). Considering the plasticity of young brain, early detection and early intervention is more effective than post-remedies (20). Therefore, it is especially important to take effective early intervention to prevent and improve neurodevelopmental outcomes in children.

Pregnant women's mental health needs more attention during the pandemic. On the one hand, SARS-CoV-2 is a neuroinvasive virus that may have direct and indirect effects on the central nervous system (9), and may have long-term adverse consequences for the patients' cognitive and neuropsychological function (9, 21). On the other hand, fear and uncertainty about the short and long-term effects on themselves and their children may deteriorate mothers' depressive and anxiety symptoms (22). Ultimately, the mental health of pregnant women may adversely affect the neurobehavioral development of newborns (23).

Breast feeding is now recommended in asymptomatic or mild symptomatic women. Nevertheless, in the first line reaction to pandemic, breast-feeding rates are very low in the first few months of the fetus, due to fear of maternal-fetal transmission (24). This may have negative effects on infant brain development and mother-infant relationship (25). Studies have found that early childhood experiences and environmental influences leave a lasting mark on brain structure and long-term health (26–28). Furthermore, early childhood adversity is related to impairment of learning, behavior, and physical and mental health in the future (27). Therefore, we advocate active health education and guidance on breastfeeding and parenting behavior for affected pregnant women and their families during the pandemic for possible mother-to-child separation and early cessation of breastfeeding on infants. Also, timely monitoring of pregnant women's mental health, so as to effectively prevent and early detect psychological trauma.

At present, there is much controversy relating to the possibility of vertical mother-to-baby transmission of SARS-CoV-2. SARS-CoV-2 has been confirmed to have the ability of passing through placental barrier (29). In addition, severe SARS-CoV-2 placenta infection may trigger fetal inflammatory response, leading to organ damage and developmental deficiencies (30). However, vertical transmission seems to occur in a minority of cases of maternal COVID-19 infection in the third trimester (31, 32). Consistent with several studies (33–36), we found no evidence of vertical transmission. Thus, we believe that there is still no definite evidence of vertical transmission of SARS-CoV-2.

Our study has drawn a link between maternal COVID-19 and early infantile developmental delay in many domains, such as communication, gross movement, problem solving, personal-social, and social-emotional. Nevertheless, there are some limitations to our study that should be considered. First, the results of this study were limited by the small sample size, which may lead to unstable results. As the first batch of affected pregnancies in Wuhan, the mothers and their newborns underwent many examinations for both clinic and research purpose, therefore the participants' participation and compliance were less optimistic than expected. However, the maternal and fetal characteristics were comparable between included and overall affected population in the current study. Second, the ASQ-3 questionnaire survey in this study is conducted by phone and the Internet. Third, whether SARS-COV-2 can be propagated through vertical mother-to-baby transmission requires further research. In the future, it is necessary to carry out follow-up studies on COVID-19 pregnant women and their newborns to address these problems in order to make up for these shortcomings and gaps.

## Conclusions

In summary, SARS-COV-2 infection during pregnancy may have an adverse effect on infant neurobehavioral development. We call for more research to estimate the burden of SARS-COV-2 infection in the formation of personality and neurobehavioral development of the children conceived in the era of the COVID-19 pandemic.

## Data Availability Statement

The data analyzed in this study is not readily available unless the application have been reviewed and approved by the Ethics Committee of Maternal and Child Health Hospital of Hubei Province. Please contact Yao Cheng (chengyao2014@sina.com) if you have any requests.

## Author Contributions

JY and YC designed the study. HT and YX drafted the manuscript. JY and HT contributed in manuscript revision. JY and MY data analysis. MY and GS contributed in data collection. All authors have read and approved the manuscript.

## Conflict of Interest

The authors declare that the research was conducted in the absence of any commercial or financial relationships that could be construed as a potential conflict of interest.

## Publisher's Note

All claims expressed in this article are solely those of the authors and do not necessarily represent those of their affiliated organizations, or those of the publisher, the editors and the reviewers. Any product that may be evaluated in this article, or claim that may be made by its manufacturer, is not guaranteed or endorsed by the publisher.
