# Corrigendum: Impact of SARS-CoV-2 Infection During Pregnancy on Infant Neurobehavioral Development: A Case-Control Study

**DOI:** 10.3389/fped.2022.853389

**Published:** 2022-03-10

**Authors:** Yao Cheng, Haoyue Teng, Yue Xiao, Mengxin Yao, Jieyun Yin, Guoqiang Sun

**Affiliations:** ^1^Department of Obstetric, Maternal and Child Health Hospital of Hubei Province, Wuhan, China; ^2^Department of Epidemiology and Health Statistics, Medical College of Soochow University, Suzhou, China

**Keywords:** COVID-19, pregnancy, infant neurobehavioral development, ASQ-3, SARS-CoV-2

In the original article, there was an error in the spelling of author name listed as Guoqing Sun. The correct name spelling is Guoqiang Sun.

In the original article, there were errors to the affiliations, an Ethical approval number was omitted, and additionally there were errors in the Methods, namely the statement of the laboratory test method for SARS-CoV-2 is not only provided by BioGerm, and the respiratory swabs included throat and nasal were collected.

In the published article, there was an error in affiliation 1. Instead of “Department of Obstetric, Maternal and Child Health Hospital, Wuhan, China,” it should be “Department of Obstetric, Maternal and Child Health Hospital of Hubei Province, Wuhan, China.”

In the **Abstract**, instead of “A case-control study was conducted in Wuhan Maternal and Child Health Hospital, China.”, it should be “A case-control study was conducted in Maternal and Child Health Hospital of Hubei Province.”

In the **Methods**, *Study Population* section, instead of “A total of 60 patients delivered at the Maternal and Child Health Hospital in Wuhan, Hubei province from January 24 to March 14, 2020, and were confirmed to have COVID-19 by laboratory and clinical diagnosis.” it should be “A total of 60 patients delivered at the Maternal and Child Health Hospital of Hubei province from January 24 to March 14, 2020, and were confirmed to have COVID-19 by laboratory and clinical diagnosis.”

In the **Methods** section, *Study Population*, instead of “This study was reviewed and approved by Medical Ethics Committee of Maternal and Child Health Care Hospital of Hubei Province.”, it should be “This study was reviewed and approved by the Ethics Committee of Maternal and Child Health Hospital of Hubei Province (No. 2021IECLW013).”

In the **Methods** section, *Data Collection*, instead of “Maternal throat swab samples were collected and tested for SARS-CoV-2 with the Chinese Center for Disease Control and Prevention (CDC) recommended Kit (BioGerm, Shanghai, China), following WHO guidelines for qRT-PCR (17).”, it should be “Maternal respiratory swabs were collected and tested for SARS-CoV-2 by reverse transcriptase–polymerase chain reaction (RT-PCR) assay (17).”

The authors apologize for these errors and state that this does not change the scientific conclusions of the article in any way. The original article has been updated.

## Publisher's Note

All claims expressed in this article are solely those of the authors and do not necessarily represent those of their affiliated organizations, or those of the publisher, the editors and the reviewers. Any product that may be evaluated in this article, or claim that may be made by its manufacturer, is not guaranteed or endorsed by the publisher.

