# Supplementary material for: Impact of SARS-CoV-2 Infection During Pregnancy on Infant Neurobehavioral Development: A Case-Control Study
Source: Front Pediatr. 2021 Dec 2;9:762684. doi: 10.3389/fped.2021.762684 (PMC8678601; doi:10.3389/fped.2021.762684)
Supplement: Supplementary file 1 [file Table_1.DOCX]

**Supplementary Table 1.** Maternal and fetal characteristics of the overall and selected patients with SARS-CoV-2 infection.

| **Variables*** | Excluded (n=51) | Included (n=9) | *P* value |
| --- | --- | --- | --- |
| ***Characteristics of pregnant women*** |  |  |  |
| Age (years) | 30.4±4.3 | 31.8±4.6 | 0.370 |
| Height (cm) | 160.6±5.6 | 162.0±4.8 | 0.511 |
| Weight (kg) | 54.8±7.7 | 57.0±4.4 | 0.430 |
| BMI (kg/m^2^) | 20.2±5.0 | 21.8±2.0 | 0.409 |
| Gravidity | 1.5 (1, 3) | 3 (3, 4) | 0.020 |
| Parity | 1 (1, 2) | 1 (1, 2) | 0.607 |
| Gestational age at delivery (week) | 39.3±2.6 | 38.0±1.1 | 0.532 |
| Delivery |  |  | 0.750 |
| Vaginal delivery | 17 (of 51) | 2 (of 9) |  |
| Caesarean section | 33 (of 51) | 7 (of 9) |  |
| Gestational hypertension | 4 (of 51) | 2 (of 9) | 0.566 |
| Gestational diabetes | 15 (of 51) | 1 (of 9) | 0.229 |
| ***Infantile characteristics*** |  |  |  |
| Male | 27 (of 51) | 3 (of 9) | 0.278 |
| Height (cm) | 49.9±1.3 | 49.2±2.1 | 0.234 |
| Weight (g) | 3246.2±484.6 | 3108.8±644.7 | 0.459 |
| Fetus number | 1.08±0.27 | 1±0.00 | 0.393 |
| Low birth weight (< 2500 g) | 3 (of 51) | 2 (of 9) | 0.102 |
| Pre-term delivery (< 37 weeks) | 9 (of 51) | 2 (of 9) | 0.744 |
| Birth defects | 2 (of 51) | 0 (of 9) | 0.537 |
| Neonatal death | 0 (of 51) | 0 (of 9) | ... |
| Fetal death or stillbirth | 0 (of 51) | 0 (of 9) | ... |
| Apgar 1 min | 9.7±0.5 | 9.8±0.4 | 0.829 |

*expressed as mean (SD) or median (interquartile range).
